# The Effect of Thermomechanical Pretreatment on the Structure and Properties of Lignin-Rich Plant Biomass

**DOI:** 10.3390/molecules25040995

**Published:** 2020-02-23

**Authors:** Ekaterina M. Podgorbunskikh, Aleksey L. Bychkov, Elena I. Ryabchikova, Oleg I. Lomovsky

**Affiliations:** 1 Institute of Solid State Chemistry and Mechanochemistry, Siberian Branch, Russian Academy of Sciences, 18 Kutateladze str., 630128 Novosibirsk, Russia; bychkov.a.l@gmail.com (A.L.B.); lomov@solid.nsc.ru (O.I.L.); 2 Novosibirsk State Technical University, 20 Karla Marksa Pr., 630073 Novosibirsk, Russia; 3 Institute of Chemical Biology and Fundamental Medicine, Siberian Branch, Russian Academy of Sciences, 8 Lavrentieva Ave., 630090 Novosibirsk, Russia; lenryab@niboch.nsc.ru

**Keywords:** thermo-mechanochemical pretreatment, lignocellulosic waste, cell wall structure, lignin-rich biomass, enzymatic hydrolysis

## Abstract

The cooperative thermomechanical properties of plant-derived polymers have been studied insufficiently, although this feedstock has a very high potential. In the present paper, we analyzed the changes in the structure and physicochemical properties of lignin-rich biomass induced by thermomechanical pretreatment. Low-temperature treatment allows one to retain the original supramolecular structure of the cell walls, while an appreciably high disintegration degree is reached. This increases the reactivity of the material in the subsequent heterogeneous reactions. Mechanical pretreatment at medium temperatures (10 °C), when almost all cell wall polymers except for low-molecular-weight lignin are in the glassy state, enhances the mobility of cell wall polymers and causes sufficient cellulose disordering, while the specific surface area is not significantly increased. High-temperature pretreatment of reed biomass is accompanied by pore formation and lignin release from the cell wall structure, which opens up new prospects for using this biomass as a matrix to produce core–shell-structured sorbents of heavy metals. The energy consumed by mechanochemical equipment for the activation of reed biomass was determined.

## 1. Introduction

Lignocellulosic materials, a common agricultural and wood industry waste, have great potential as renewable feedstock that can be converted into platform chemicals, second-generation biofuels, and a number of other high-demand chemical products [1]. Integrated biorefinery, which comprises the conversion of polysaccharides to low-molecular-weight carbohydrates [2,3,4] and the use of lignin as a component of high-energy fuel [5,6,7] or core–shell-structured sorbents with humic acids [8], is the most promising green approach in the refinery of plant biomass. Because of its complex supramolecular structure, the lignocellulosic biomass requires mechanical [9,10,11], thermal [12,13,14] or chemical pretreatment [15,16,17]. The properly selected pretreatment method has a favorable effect on the efficiency of subsequent processes, since it modifies the material structure, enhances the availability of the target components and the specific surface area of particles, as well as reduces cellulose crystallinity.

Special attention should be paid to the structure and properties of lignin, whose content in the cell wall can be as high as 10%–45% [18,19,20]. In a number of cases, lignin or its degradation products may shield the surface from the reagents after pretreatment, and the total reactivity will be low even if there are a well-developed specific surface area (Sspec = SSA) and low crystallinity of cellulose [18,19,20].

The phase transition temperatures for individual polymers are known. The melting point and melt flow temperature lie in a broad range (90–193 °C) [21,22,23]. Cellulose is characterized by the glass transition temperature of −27 °C, the melting point of 135 °C, and the melt flow temperature of 240–244 °C [21,24]. The melting points of hemicelluloses vary significantly depending on the type of plant biomass, but are not higher than 167–181 °C [21].

However, the thermomechanical characteristics of native plant materials have been insufficiently covered in the literature and differ from those of individual extracted components. In some cases, woody lignocellulosic materials can exist in a glassy state at temperatures as high as 16 °C [24]. Melting of the most refractory fraction of hemicellulose occurs at temperatures above 180 °C [21], being accompanied by the decomposition of the lignocellulosic material.

This study was aimed at investigating the physicochemical and structural modifications caused by thermomechanical pretreatment of lignin-rich biomass.

## 2. Results and Discussion

Stem biomass of common reed (Phrágmites austrális) was used as biomass to study the effect of mechanical pretreatment on cell wall polymers in lignin-rich materials. Over the past years, common reed has been widely used as feedstock to produce second-generation biofuel and as a material for the construction and chemical industries [25,26].

Stem biomass of common reed contains cellulose, 22.9% ± 0.2% (evaluated using the Kushner’s method); pentosanes, 20.6% ± 0.3%; acid-insoluble lignin (Klason lignin), 40.2% ± 0.4%; extractives, 11.1% ± 0.5%; and ash, 3.3% ± 0.1%. This material can be classified as a lignin-rich material, so it is a convenient object to study the phase transitions of lignin.

**Figure 1:**
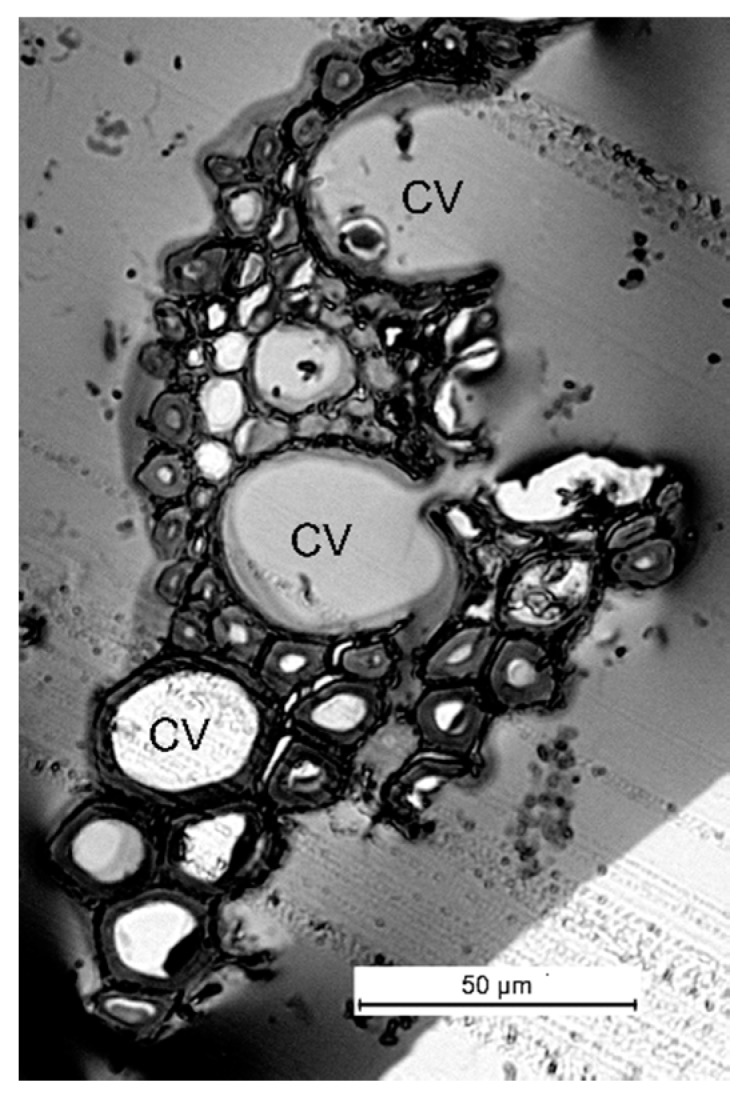
A microimage of the transverse cross-section of the initial reed stem specimen (after pre-milling on a cutting mill; a semithin cross-section; light optical microscopy): CV—conducting veins.

The native tissue of reed stems mainly consists of the phloem and the xylem, with a high content of large and small vascular bundles (Figure 1) surrounded by guard cells. The tissue morphology is consistent with the findings reported previously [27]. The cells are different sizes but have a similar round shape. One can clearly see that the tissue is disintegrated along large vascular bundles during the pre-milling.

**Figure 2:**
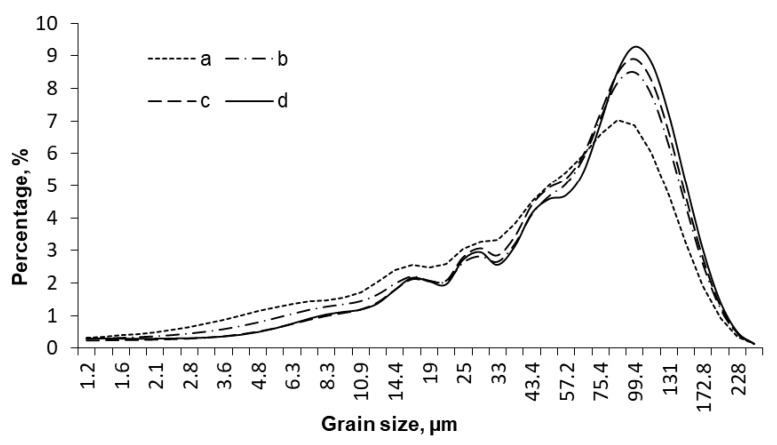
Grain size distribution in the products obtained by mechanical treatment at −196 °C (a), 10 °C (b), 100 °C (c) and 180 °C (d).

The phase composition of structure-forming polymers has a crucial effect on biorefinery efficiency. The migration of macromolecules across the material depends on pretreatment conditions and is largely responsible for the characteristics of the final product. In order to qualitatively analyze the efficiency of specimen grinding at different temperatures, we measured the grain size distribution profiles by laser diffraction scattering (LDS) (Figure 2).

The most significant reduction in grain size was observed for brittle destruction of the specimen pretreated at a boiling point of liquid nitrogen, when all the components exist in the glassy state. The higher pretreatment temperature converts grinding to the plastic-strain mode, thus reducing grinding efficiency and causing smaller changes in the average grain size of the material.

The specific surface area of the resulting products typically increases not only due to disintegration but also due to the formation of cracks and open pores. These effects were studied by nitrogen desorption using the Gregg and Sing approximation.

The brittle grinding mode implemented when the mill reactor is thermostated in liquid nitrogen and all the biomass components exist in the glassy state allows one to significantly reduce the average grain size and increase the specific surface area to 4.3 m^2^/g (Table 1). Mechanical treatment at 10 °C is accompanied by sequential polymer devitrification, which explains why Sspec was reduced to some extent compared to the findings obtained at −196 °C. At 100 °C and 180 °C, specimen disintegration proceeds in the plastic mode; the polymers become highly elastic. They have a high specific surface area (up to 5.0 m^2^/g), which will be attributed to partial lignin decomposition and formation of a large number of pores in the text below.

Pretreatment using the brittle disintegration mode leads to efficient amorphization of crystalline regions in cellulose (Table 1). The devitrification temperature of cellulose and hemicelluloses is reached as the pretreatment temperature is increased, which explains why more energy is consumed in the case of plastic deformation and amorphization becomes less efficient.

Native cellulose I exists in two polymorphic modifications: Iα and Iβ. The high temperature of mechanical treatment and the local overheating points at the interface between the biomass and grinding bodies may cause partial recrystallization of metastable cellulose Iα into the more stable cellulose Iβ, which can explain why the crystallinity indices of the specimens slightly changed after mechanical pretreatment at 100 and 180 °C [28,29].

**Figure 3:**
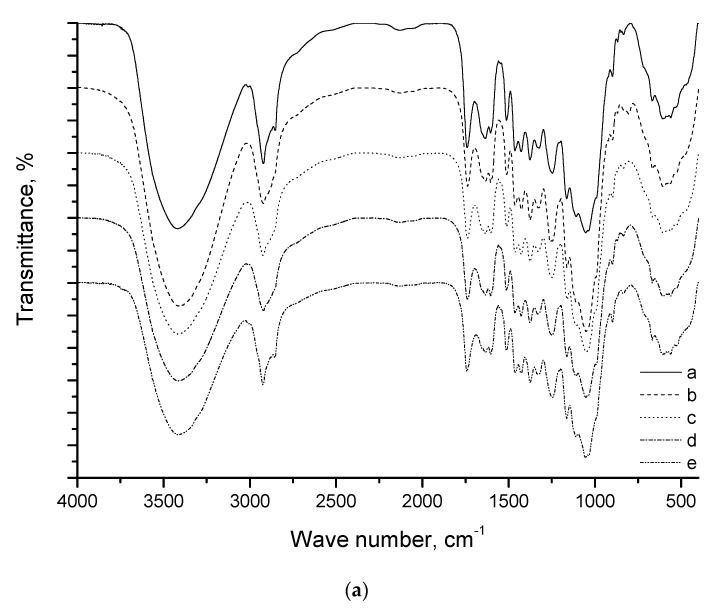

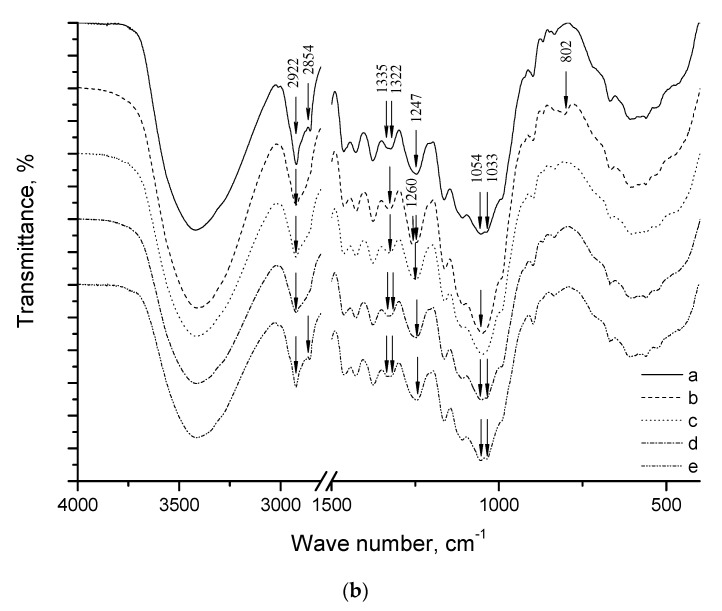
(**a**) IR spectra of the initial reed biomass (a) and the products of mechanical pretreatment at −196 °C (b), 10 °C (c), 100 °C (d), and 180 °C (e); (**b**) detail of the 4000–2800 cm^−1^ and 1500–400 cm^−1^ range.

The infrared spectra of the native reed biomass are a superposition on the characteristic bands of all cell wall polymers (Figure 3) and contain all phenylpropane structural monomeric units of lignin (guaiacyl (G), syringyl (S), and *p*-hydroxyphenyl (H) units) that are typically found in herbaceous plants. The spectra of the reed biomass contain stretching and bending vibrations of aromatic and aliphatic OH groups (at 3415 and 1163 cm^−1^, respectively). The bands at 2922 and 2854 and 1464 cm^−1^ are ascribed to asymmetric and symmetric vibrations of methylene and methyl (CH_2_ + CH_3_) groups. The bands at 1604, 1518 and 1428 cm^−1^ correspond to aromatic ring vibrations of the phenylpropane skeleton.

The intensity of deformation vibrations of adsorbed water (1640 cm^−1^) is associated with moisture content. The intensity of the band corresponding to symmetric stretching vibrations of C–H bonds (2854 cm^−1^) decreases and the band acquires a shoulder shape after mechanical treatment at −196, 10, and 100 °C. A hypsochromic shift of carbonyl group (>C=O) in hemicelluloses (acetyl groups of hemicelluloses) and lignin (aldehyde groups in lignin) is observed under the same conditions, thus demonstrating that structural changes take place in the lignin–hemicellulose matrix.

With low-temperature treatment (−196 and 10 °C), the bands of 1322 cm^−1^ (S) and 1033 cm^−1^ (G) disappear. The sample treated at −196 °C shows a band of 1260 cm^−1^, which determines acetyl groups in hemicelluloses. For the same sample, the intensity of the bands of 1107 cm^−1^ (S) and 1054 cm^−1^ (G) increases. A band of 800 cm^−1^ is related to the vibrations of the glucopyranose ring associated with pendular vibrations of C–H and C–H_2_ bonds.

**Figure 4:**
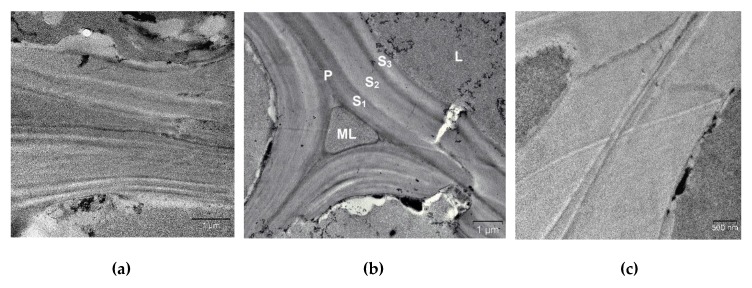
The cell walls of the initial reed biomass (TEM, ultrathin cross-sections): ML—the middle lamella; P—the primary cell wall; S1, S2, and S3—the secondary cell wall layers; and L—the intracellular substance; (**a**), (**b**) and (**c**)—different regions of sample.

Thermomechanical pretreatment of the biomass is accompanied by significant modification of the supramolecular structure. Figure 4 shows the microimages of ultrathin cross-sections of the biomass. The cell walls vary in shape and thickness; their ultrastructure corresponds to that earlier described in the literature [30].

The electron-dense layers are rich in lignin and hemicelluloses, while the electron-transparent ones are rich in cellulose. One can also see the intracellular space and pores with the contents characterized by different electron densities.

**Figure 5:**
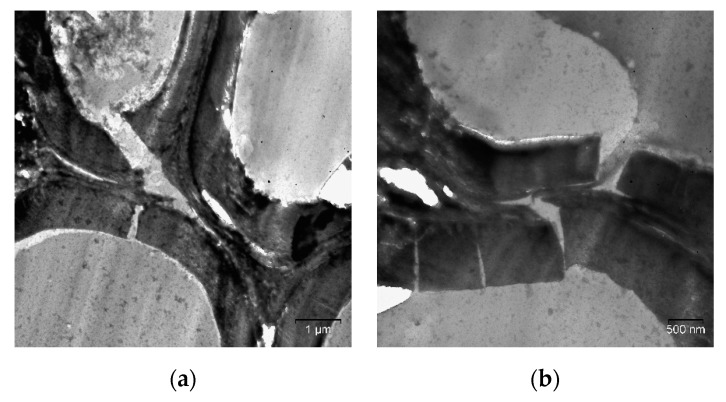
Cell walls of the reed biomass after mechanical pretreatment at −196 °C (TEM, ultrathin cross-sections): (**a**) the general view of the fragmented cell walls; (**b**) cell wall fragments.

Mechanical pretreatment at the boiling point of liquid nitrogen leads to cell wall fragmentation owing to brittle disintegration (Figure 5). Cracks are formed in the direction perpendicular to the cell wall, and longitudinal cracks are subsequently formed.

**Figure 6:**
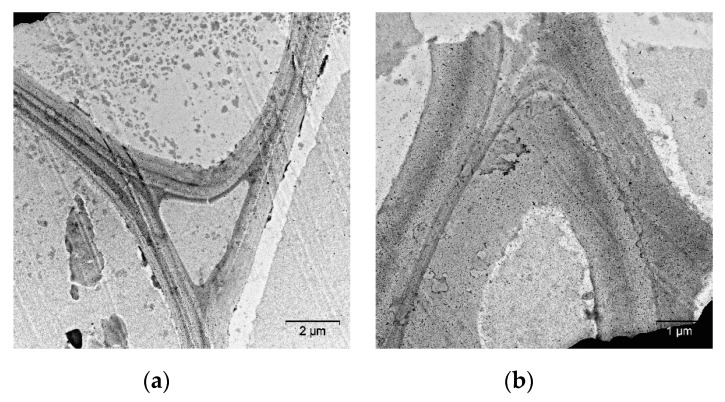
TEM images of ultrathin cross-sections of reed biomass after mechanical pretreatment at 10 °C: (**a**) the general view of cell walls; (**b**) cell wall fragments.

The supramolecular structure of cell walls was gradually disordered as the temperature of mechanical pretreatment was increased to 10 °C (Figure 6). The regular layered structure was disrupted, and the electron density of the layers decreased.

**Figure 7:**
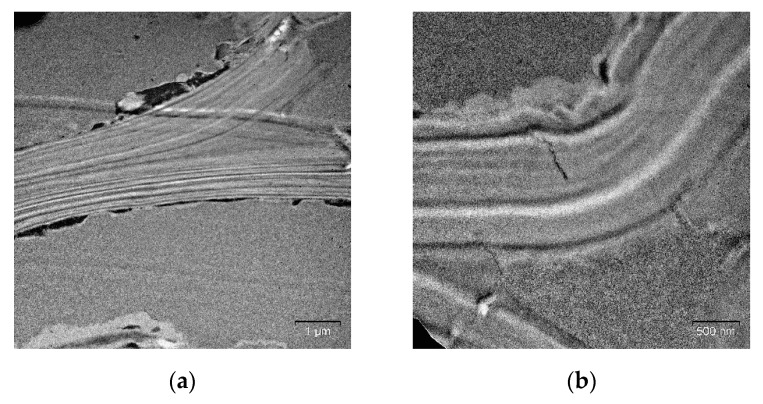
Cell walls of the reed biomass after mechanical pretreatment at 100 °C (TEM, ultrathin cross-sections): (**a**) general view of the disordered cell wall; (**b**) the magnified view of a cell wall fragment.

**Figure 8:**
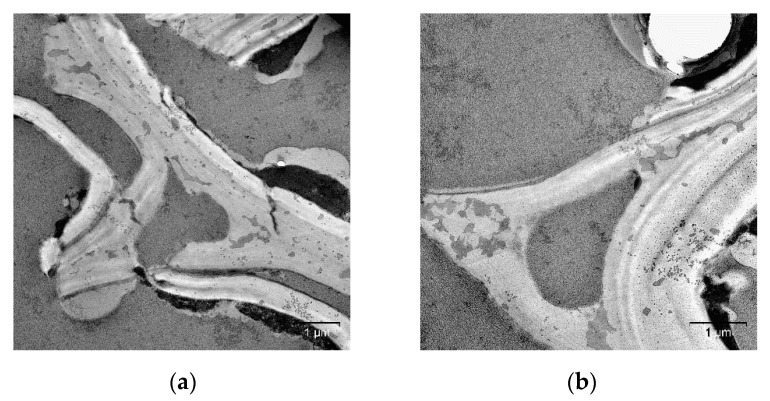

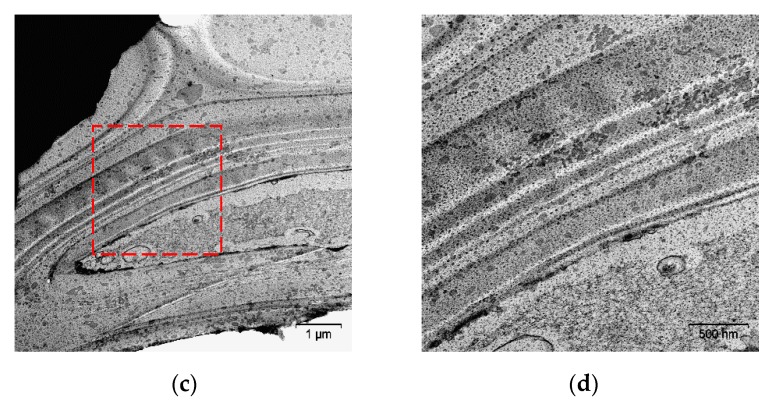
Cell walls of the reed biomass after mechanical pretreatment at 180 °C (TEM, ultrathin cross-sections): (**a**,**b**) the general view of the disordered cell wall; (**c**,**d**) cell wall fragments.

Mechanical pretreatment at 100 °C led to the disruption of cell walls and electron density layers: they were displaced with respect to each other (Figure 7), which can probably be explained by the rupturing of intra- and intermolecular hydrogen bonds between the cell wall components after the thermomechanical exposure. Further elevation of the temperature of mechanical pretreatment to 180 °C caused deep structural disordering in cell walls and the formation of an electron-dense substance on the surface (Figure 8), which appears to be pseudo-lignin (lignin and its decomposition products) [31,32].

Mechanical pretreatment at 180 °C yields a large number of pores with a diameter ranging from 10–20 nm to 500–200 nm, which explains why the specific surface area that can be measured by thermal desorption of nitrogen has increased (Table 1).

Enzymatic hydrolysis was performed to elucidate the relationship between surface properties and the reactivity of mechanically pretreated specimens. This process is a good method for characterizing the changes, since it is a combination of heterogeneous reactions that depend on the surface composition, specific surface area, and the crystallinity index.

Brittle disintegration of material at −196 °C significantly increases both the initial rate of hydrolysis (1.8-fold) and its yield (by 6.0%) as the crystallinity index is reduced (by 12%) and a new accessible surface (4.3 m^2^/g) is formed.

The specimens obtained at high temperatures (100 and 180 °C) are the least reactive ones. A layer of lignin and products of its partial decomposition is formed under these conditions on the surface of material grains having the highest specific surface area (Figure 8). Along with the low degree of cellulose amorphization, it impedes the enzymatic hydrolysis reactions: the hydrolysis yield increases from 13.3% to 16.2% and 14.7% for the specimens pretreated at 100 and 180 °C, respectively.

The maximum rise in the initial hydrolysis rate (1.9-fold) and yield (from 13.3% to 21.2%), while the crystallinity degree declines by 11% and specific surface area decreases to 2.6 m^2^/g, corresponds to mechanical activation of the biomass at medium temperatures (10 °C). The key rate-limiting process, lignin redistribution from the cell wall to the surface, does not occur under these conditions.

**Figure 9:**
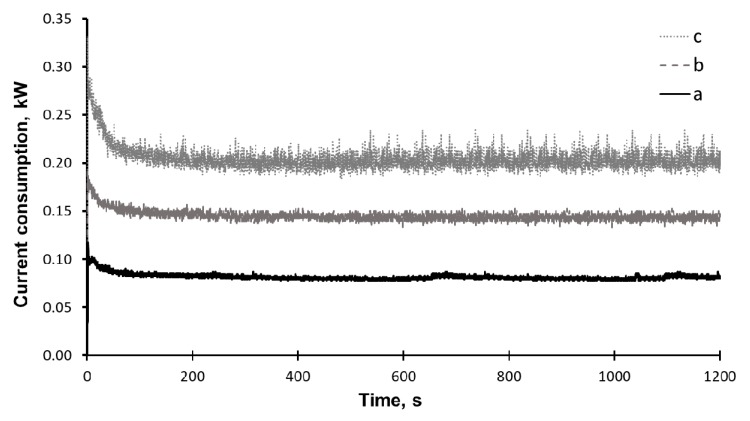
Current consumption laboratory-scale attritor (a) drove with empty jar, (b) jar with grinding bodies, and (c) jar with grinding bodies and common reed.

The current power consumed by the laboratory-scale attritor and the total energy spent on activation were recorded using a high-speed wattmeter. Figure 9 shows the current consumption changes dynamics during the operation of the mechanical equipment in various conditions. The total energy expended on the mechanical activation of reed biomass for 20 min is estimated at 86.6 kWh.

Due to the porous structure and the layer of lignin-like compounds formed on the particle surface during high-temperature mechanical pretreatment, this material is promising for designing core–shell-structured sorbents of heavy metals. In these sorbents, humic acids from the “shell” will be stabilized on the surface of the “core” coated with polyphenolic molecules of lignin and its degradation products.

## 3. Materials and Methods

The following reagents and materials were used in this study: common reed (*Phrágmites austrális*) biomass (collected in 2017 in the Novosibirsk region, Russia; coordinates: 55°1.8119′0″ N, 82°55.2258′0″ E), sodium azide (99%, Sigma-Aldrich, St. Louis, MO, USA), sodium acetate (99%, Sigma-Aldrich), potassium hexacyanoferrate(III) (99%, Sigma-Aldrich), D(+)-glucose (99%, Sigma-Aldrich, St. Quentin Fallavier, France), acetic acid (99%, Sigma-Aldrich), and CelloLux 2000 enzyme cocktail (Production Enterprise OOO Sibbiopharm Ltd., Berdsk, Novosibirsk region, Russia). The CelloLux A enzyme cocktail was a mixture of enzymes exhibiting biocatalytic activity immobilized on the model substrates: xylanase (8000 U/g); cellulase (2000 U/g); β-glucanase (up to 1500 U/g), and glucoamylase (20 U/g).

Enzymatic hydrolysis was carried out at 50 °C in a thermostated shaker at a shaking speed of 120 rpm in 1:20 acetate buffer (pH 4.6). The reaction mixture was preserved with sodium azide. The reducing sugar content was measured using the Hagedorn–Jensen ferrocyanide method.

Granulometric analysis was conducted by laser diffraction scattering (LDS). The specific surface area of the specimens was determined according to thermal desorption of nitrogen. The cellulose crystallinity index was measured on an X-ray diffractometer (CuKα radiation, Bregg–Brentano geometry in the reflection mode). The IR spectra were recorded as 100 scans with a Vector-22 IR-Fourier spectrometer (Bruker) in KBr pellets (4 mg of the specimen per 540 mg of KBr) in the wavelength range of 400–4000 cm^−1^ with a resolution of 2 cm^−1^.

Klason (acid-insoluble) lignin and extractives were quantified pursuant to the TAPPI T222 om-02 and TAPPI T 204 cm-07 test methods. The content of cellulose referred to as Kürschner cellulose was determined via isolation using a 4:1 (*v*/*v*) mixture of nitric acid and ethanol for 4 h. Pentosans were transformed to furfural in boiling 13% hydrochloric acid and determined spectrophotometrically using orcinol-ferric chloride reagent according to the T 223 cm-01 test method. The ash content was measured at 525 °C according to the TAPPI T 211 om-02 test method.

Mechanical pretreatment of the biomass was conducted in a laboratory-scale attritor (manufactured at the Institute of Solid State Chemistry and Mechanochemistry, SB RAS, Novosibirsk, Russia) filled with steel grinding bodies (9 mm in diameter) and equipped with a thermostat system. The temperatures of the thermostat circuit were −196, 10, 100, and 180 °C. The treatment duration was 20 min and the rotor speed was 600 rpm.

The energy costs for machining were measured using a Mercury high-speed wattmeter (Incotex Electronics Group, Russia) connected to the DVP-SA2 industrial controller (Delta Electronics, Inc., Taipei, Taiwan) using the ModBus protocol.

Transmission electron microscopy. The specimens were fixed with 1% gelatin solution and then immobilized in 1% osmium tetroxide solution, dehydrated using a series of ethanol solutions of increasing concentrations and acetone, and embedded in an Epon–Araldite mixture. Semithin and ultrathin cross-sections were prepared using a Leica EM UC7 ultramicrotome (Leica, Wetzlar, Germany).

The semithin cross-sections were stained with 1% azure II solution. The ultrathin cross-sections were examined without additional contrasting. The specimens were studied on an Axioimager Z light microscope (Carl Zeiss, ‎Oberkochen, Germany) and a JEM 1400 transmission electron microscope (Jeol, Tokyo, Japan) at an accelerating voltage of 80 kV. The images were recorded using a Leica DFC420 C digital camera (Leica, Germany) and a Veleta side-mounted camera (EMSIS, Muenster, Germany).

## 4. Conclusions

Thermomechanical pretreatment significantly alters the physicochemical properties and structure of lignin-rich plant biomass.

Low-temperature pretreatment allows one to retain the initial supramolecular structure of cell walls, thus ensuring a significant disintegration degree and increasing material reactivity during subsequent heterogeneous hydrolysis.

Mechanical pretreatment at medium temperatures (10 °C), when almost all cell wall polymers except for low-molecular-weight lignin exist in the glassy state, makes cell wall polymers more mobile. Specific surface area does not increase substantially under these conditions, but cellulose becomes disordered to an appreciable extent.

High-temperature pretreatment of reed biomass is accompanied by pore formation and lignin release from the cell wall structure, thus opening up new prospects for using this biomass as a matrix to produce core–shell-structured sorbents of heavy metals.

## Figures and Tables

**Table 1:** Specific surface area and crystallinity indices of the initial and mechanically treated reed biomass.

Specimen	Average Grain Size, µm	Sspec, m^2^/g	Crystallinity Index, %
Initial reed biomass	0.5–1 mm	0.5 ± 0.1	72 ± 3
Mechanical treatment at −196 °C	45 ± 4	4.3 ± 0.3	60 ± 4
Mechanical treatment at 10 °C	54 ± 4	2.6 ± 0.2	61 ± 4
Mechanical treatment at 100 °C	65 ± 6	4.1 ± 0.3	66 ± 4
Mechanical treatment at 180 °C	63 ± 5	5.0 ± 0.3	67 ± 5
